# Clinical values of the early detection of serum procalcitonin, C-reactive protein and white blood cells for neonates with infectious diseases

**DOI:** 10.12669/pjms.326.11395

**Published:** 2016

**Authors:** Shiwen Liu, Yunxiu Hou, Haili Cui

**Affiliations:** 1 Shiwen Liu, Central Laboratory, Binzhou Medical University Hospital, Shandong, 256603, China; 2 Yunxiu Hou, Health Management, Binzhou Medical University Hospital, Shandong, 256603, China; 3 Haili Cui, Central Laboratory, Binzhou Medical University Hospital, Shandong, 256603, China

**Keywords:** Newborn, C-reactive protein, Procalcitonin, Infectious disease

## Abstract

**Objective:**

To discuss application values of serum procalcitonin (PCT), C-reactive protein (CRP) and white blood cells (WBC) count in early diagnosis and treatment of neonatal bacterial infectious diseases.

**Methods:**

Clinical data of one hundred and thirty-six newborns with infectious diseases who were admitted into the hospital were retrospectively analyzed. They were divided into bacterial infection group (N=70) and non-bacterial infection group (N=66). Additionally, sixty-six healthy newborns who underwent physical examination in our hospital in the same period were selected as controls. Subjects in the three groups were all detected for serum PCT, CRP and WBC levels.

**Results:**

The levels of PCT, CRP and WBC in the bacterial infection group were much higher than those of the non-bacterial infection group and the healthy control group, and the differences had statistical significance (P<0.05). The positive rates of PCT, CRP and WBC of the bacterial infection group were higher than those of the non-bacterial infection group (P<0.05); the specificity and sensitivity of the PCT level were obviously higher than those of the CRP and WBC levels in diagnosing bacterial infectious diseases (P<0.05).

**Conclusion:**

Serum PCR, CRP and WBC levels are of high diagnostic values to neonatal infectious diseases. Compared to WBC and CRP, PCT is more sensitive index in the diagnosis of neonatal infectious diseases.

## INTRODUCTION

Neonatal infectious diseases which are common in pediatric clinics refer to inflammatory diseases induced by pathogenic microorganism in neonatal period. Neonatal infectious diseases featured by sudden onset, rapid development and severe disease condition are of high risks to cause death.^1^,^2^ Neonatal period is a special period during the development of body; however, many infectious diseases are seen in this period, which may be because newborns have poor regulation ability and weak immune barrier and invasive operations such as invasive ventilators and deep vein catheterization are easy to induce nosocomial infection during emergency treatment of newborns.^3^ Therefore, the early diagnosis and timely treatment of infectious diseases is of positive significance to improve diagnostic and treatment levels and reduce the fatality rate of newborns.

The major pathogenic bacteria of neonatal infectious diseases include viruses, bacteria, atypical pathogens, parasites and fungi, among which, bacteria and viruses are the most common. In clinics, different pathogenic infections can manifest similar clinical symptoms, which makes diagnosis difficult; the clarity of pathogenic bacteria is beneficial to early treatment and the improvement of curative effect.^4^ Currently, there are many indexes for evaluating infectious diseases. The detection of white blood cell count is a major traditional approach for identifying bacterial infection; however, its sensitivity and specificity in diagnosing bacterial infection are low.^1^ C-reactive protein (CRP), a kind of acute phase reactive protein synthesized by the liver under stress state, can regulate inflammatory reaction and defense infectious diseases.^5^,^6^ A recent study demonstrated that, serum procalcitonin (PCT) level can help early identify bacterial and non-bacterial infection and moreover could positively guide the clinical use of antibiotics.^7^ This study investigated the application values of serum PCT, PCR and WBC in the early diagnosis of neonatal bacterial infectious diseases by analyzing their levels.

## METHODS

### Research subjects

Clinical data of one hundred and thirty-six children who were diagnosed as infectious diseases in our hospital from Feb. 2013 to Feb. 2015 were collected and retrospectively analyzed. The children were divided into a bacterial infection group (N=70) and a non-bacterial infection group (N=66) according to clinical manifestations and laboratory detection results. Additionally, 66 healthy newborns who were born in the same hospital were selected as controls.

### Exclusion and inclusion criteria

Newborns who took antibiotics, glucocorticoids or blood products such as human immunoglobulin, plasma, red blood cells and albumin before admission or whose mother had severe pregnancy complications were excluded. Patients who were confirmed as infectious diseases according to the criteria of Practical Neonatology (4^th^ edition) and whose patients signed informed consent were included.

### Methods

Serum PCT, CRP and WBC levels of all patients and healthy newborns were measured. Firstly, 2 ml of fasting venous blood was extracted from every subject in the morning and then centrifuged to separate serum. The serum PCT level was detected with semi-quantitative colloidal gold immunobinding assay method using a LUMI test PCT detection reagent instrument (Brahms Diagnostica Company, Germany) and matched reagents; a PCT level of 0.5 μg·L^-1^ meant a positive result. The serum CRP level was detected with immune scatter turbidimetry using a Beckman IMMAGE instrument and matched reagents; a CRP level higher than 5. 2 mg·L^-1^ meant a positive result. The WBC count was detected using a Sysemx XT-1800i blood analyser (Sysemx Company, Japan); a WBC level higher than 10×10^9^/L indicated a positive result.

### Statistical analysis

SPSS ver. 20.0 was used for statistical processing. The detection results of all groups were expressed as mean±SD and analyzed by t test. Categorical data were expressed as percentage and processed by Chi-square test. The sensitivity and specificity of various indexes in the diagnosis of infectious diseases were determined based on receiver operating characteristic curve (ROC). Difference was considered statistically significant if P<0.05.

## RESULTS

### Comparison of general data between different groups

In the bacterial infection group (n=70), there were 38 males and 32 females, aged 0~28 days (average 10.81±8.42 days) and weighed 2.5 kg to 4.0 kg (average 3.56±0.60 kg). In the non-bacterial infection group (n = 66), there were 34 males and 32 females, aged 1~27 days (average 9.03±7.09 days) and weighed 2.3 kg to 3.8 kg (average 3.18±0.65 kg). The sex, age and birth weight of newborns in the three groups had no significant differences (P>0.05); hence the results were comparable.

### The comparison of CRP, PCT and WBC levels between groups

The CRP, PCT and WBC levels of the patients in the bacterial infection group were higher than those of the non-bacterial infectious diseases and the control group, and the difference had statistical significance (P<0.05); the CRP, PCT and WBC levels of the patients in the non-bacterial infection group were higher than those of subjects in the control group, and the difference were statistically significant (P<0.05) (Table-I).

**Table-I:** Comparison of serum CRP, WBC and PCT levels between the three groups (mean±SD).

Group	PCT(ng/mL)	CRP(mg/L)	WBC(×10^9^/L)
Bacterial infection group	4.03±0. 84*^#^	22.21±3.24*^#^	13.47±3.07*^#^
Non-bacterial infection group	1.16±0.48*	9.12±2.08*	8.49±3.88*
Control group	0.15±0.30	2.47±1.84	7.36±2.24

# ***Note:*** indicated P < 0.05, compared to the non-bacterial infection group;

* indicated P<0.05, compared to the control group.

### Comparison of the positive rates of CRP, PCT and WBC between the bacterial infection group and the non-bacterial infection group

The positive rates of the PCT, CRP and WBC levels of the bacterial infection group were higher than those of the non-bacterial infection group, and the differences were statistically significant (P<0.05), as shown in Table-II.

**Table-II:** Comparison of the positive rates of CRP, PCT and WBC between the bacterial infection group and the non-bacterial infection group [N(%)].

Group	Positive rate of PCT	Positive rate of CRP	Positive rate of WBC
Bacterial infection group (N=70)	67(95.7)	69(98.6)	65(92.9)
Non-bacterial infection group (N=66)	8(12.1)	11(16.7)	3(4.5)
X^2^	18.417	13.352	20.379
P	<0.05	<0.05	<0.05

### Comparison of the sensitivity and specificity of serum PCT, CRP and WBC levels in the diagnosis

**Fig.1:**
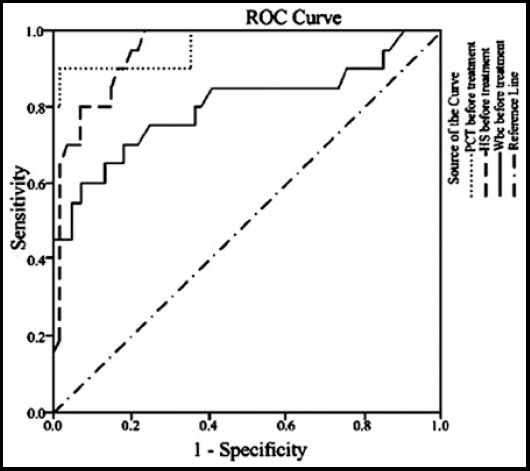
Diagonal segments are produced by ties.

In the bacterial infection group, the performance of PCT, CRP and WBC levels in the diagnosis of neonatal infection before antibiotic treatment, i.e., in the early stage of infection, was evaluated using ROC. Before treatment, the area under ROC of PCT was 0.967, higher than that of CRP (0.916) and WBC (0.805), suggesting PCT was the most valuable in the early diagnosis of neonatal infectious diseases (Fig.1). It was found that, the sensitivity, specificity, positive predicted value and negative predicted value of PCT level were much higher than those of CRP and WBC levels (P<0.05). Details are shown in Table-III.

**Table-III:** Comparison of the sensitivity and specificity of serum PCT, CRP and WBC levels in diagnosis.

Index	Cut-off point	Sensitivity	Specificity
PCT(ng/mL)	1.99	90.3%	98.1%
CRP(mg/L)	6.75	82.4%	77.2%
WBC(×10^9^/L)	14.54	81.2%	64.3%

## DISCUSSION

Infectious diseases as the key diseases in pediatrics are mainly induced by bacteria, virus and parasite.^8^,^9^ Diseases such as septic shock induced by bacterial infection are independent risk factors for the death of newborns. Hence searching for a high-efficient and effective laboratory method is of great clinical significance to the diagnosis of bacterial infection.^10^

This study provides a scientific guidance for the clinical diagnosis and treatment of pediatric infectious diseases of newborns through investigating the values of serum PCT, CRP and WBC levels in the diagnosis of pediatric infectious diseases. Research results demonstrated that, the serum PCT, CRP and WBC levels of the bacterial infection group and the non-bacterial infection group were remarkably higher than those of the control group and moreover the levels of the bacterial infection group were the highest. WBC is one of the commonly seen inflammatory markers in clinics. In pathological state, WBC level can show a remarkable increase or decrease due to different immunoreactions states and pathogenic bacteria, both of which indicated systemic bacterial infection.^11^,^12^ CRP level is one of the frequently used indexes for the diagnosis of infection currently in China.^13^ In normal state, serum CRP level is so low that it nearly cannot be detected; however, in the pathological state of infection, especially in acute stage, endogenous transmitters released by WBC accelerate hepatocyte to synthesize CRP in 4~6 hours after the occurrence of infection, which results in the sudden increase of CRP level in serum; in 36~50 hours after the occurrence of infection, serum CRP level reaches the peak value which is 100~1000 times as high as normal value.^14^ PCT, a kind of glycoprotein without hormone activity, is a calcitonin propeptide substance composed of 116 amino acids. The PCT level of healthy people or patients with non-bacterial infection is extremely low; but once human body is infected with bacteria, different tissues and organs will release a large amount of PCT.^15^,^16^

The research results demonstrated that, the combined detection of serum PCT, CRP and WBC could be taken as a clinical method for identifying neonatal infectious diseases. But the specificity of the serum CRP level in the diagnosis of bacterial infection was not ideal. Marik et al.^17^ found that, CRP as a non-specific inflammatory index also had a high level in patients with viral infection, malignant tumors, severe trauma and autoimmune disease, besides in patients with bacterial infection. WBC has low specificity and sensitivity as it is easy to be influenced by non-infectious factors such as sports, emotion and physiologic peak.^18^

This study found that, the positive rate of PCT in the bacterial infection rate was much higher than that of CRP and WBC, and the difference had statistical significance (P<0.05). It indicated that, PCT was more sensitive than CRP and WBC in the early stage of bacterial infection, which was consistent with the findings of She et al.^19^

Besides, the research results demonstrated that, serum PCT, CRP and WBC levels showed high sensitivity and specificity in the diagnosis of bacterial infection diseases; PCT level especially had a sensitivity and specificity higher than 85%, higher than the sensitivity and specificity of serum CRP and WBC levels. It further suggested the advantages of serum PCT level in the diagnosis of infectious diseases.

## CONCLUSION

In conclusion, the detection of PCT, CRP and WBC levels can provide valuable reference for the early accurate diagnosis, effective and reasonable treatment and favorable prognosis of neonatal bacterial infection. Compared to other biomarkers, PCT as a novel inflammatory index has a high value in identifying bacterial infection and it is an ideal biomarker in distinguishing bacterial and non-bacterial infection.
